# Image-guided cancer surgery: a narrative review on imaging modalities and emerging nanotechnology strategies

**DOI:** 10.1186/s12951-023-01926-y

**Published:** 2023-05-18

**Authors:** Barbara Bortot, Alessandro Mangogna, Giovanni Di Lorenzo, Guglielmo Stabile, Giuseppe Ricci, Stefania Biffi

**Affiliations:** 1 Obstetrics and Gynecology, Institute for Maternal and Child Health, IRCCS Burlo Garofolo, Trieste, Italy; 2 Department of Medicine, Surgery and Health Sciences, University of Trieste, Trieste, Italy

**Keywords:** Image-guided surgery, Cancer, Optical imaging, Intraoperative ultrasound, Radio-guided surgery, Nanoparticles, Tumor targeting

## Abstract

Surgical resection is the cornerstone of solid tumour treatment. Current techniques for evaluating margin statuses, such as frozen section, imprint cytology, and intraoperative ultrasound, are helpful. However, an intraoperative assessment of tumour margins that is accurate and safe is clinically necessary. Positive surgical margins (PSM) have a well-documented negative effect on treatment outcomes and survival. As a result, surgical tumour imaging methods are now a practical method for reducing PSM rates and improving the efficiency of debulking surgery. Because of their unique characteristics, nanoparticles can function as contrast agents in image-guided surgery. While most image-guided surgical applications utilizing nanotechnology are now in the preclinical stage, some are beginning to reach the clinical phase. Here, we list the various imaging techniques used in image-guided surgery, such as optical imaging, ultrasound, computed tomography, magnetic resonance imaging, nuclear medicine imaging, and the most current developments in the potential of nanotechnology to detect surgical malignancies. In the coming years, we will see the evolution of nanoparticles tailored to specific tumour types and the introduction of surgical equipment to improve resection accuracy. Although the promise of nanotechnology for producing exogenous molecular contrast agents has been clearly demonstrated, much work remains to be done to put it into practice.

**Graphical Abstract:**

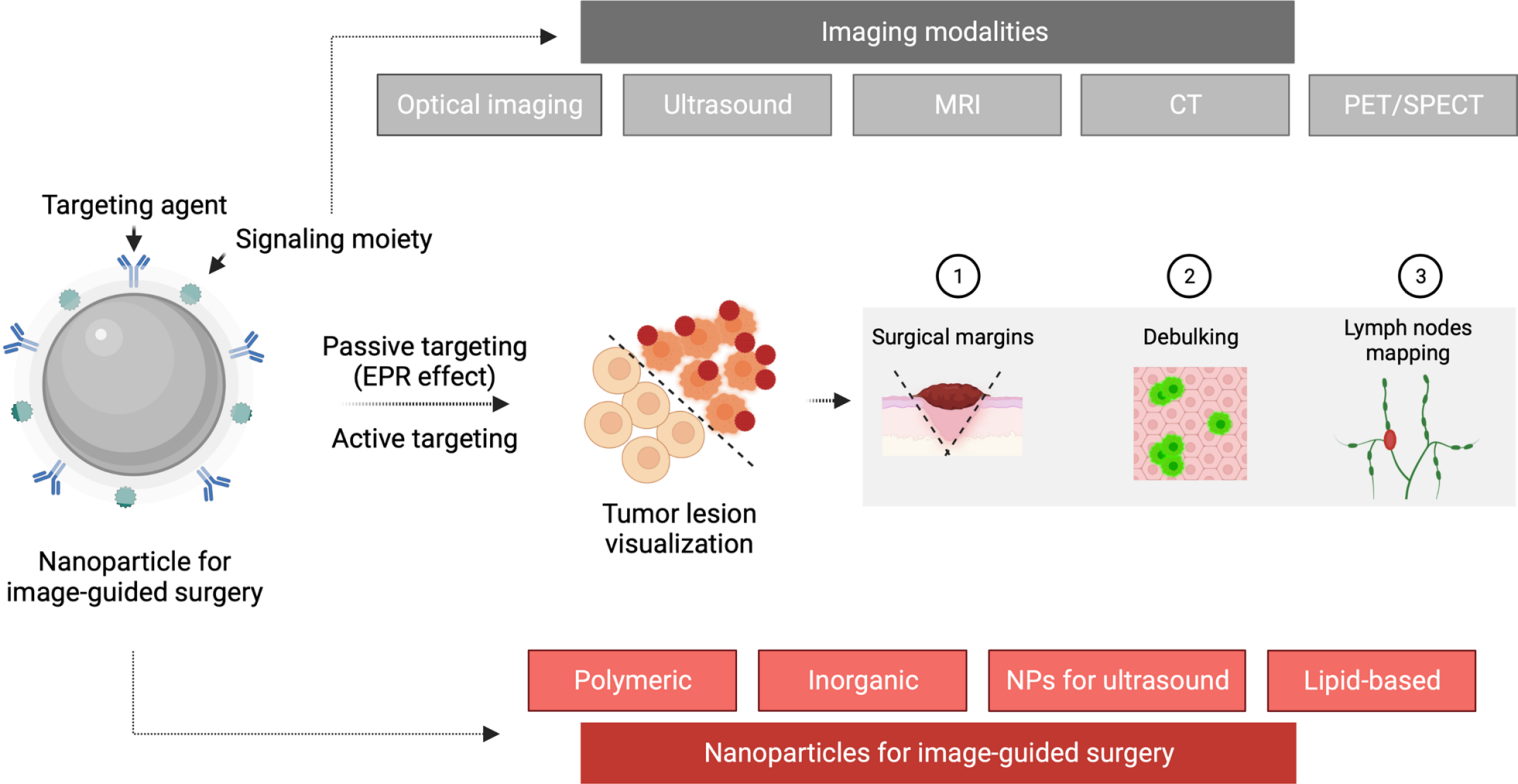

## Introduction

Cancer is a substantial contributor to the global illness burden. In 2019, there were an expected 23.6 million new cancer diagnoses and 10.0 million cancer deaths worldwide [1]. New technology, research, and innovation in cancer diagnostics and therapeutics have been advocated to improve this high impact, leading to current efforts such as the Precision Medicine Initiative in January 2017 [2]. Although molecularly targeted therapies have resulted in significant improvements in survival outcomes, the surgical treatment to achieve complete resection of tumour lesions remains an integral part of the cure for most solid tumours and one of the most important prognostic factors for patient survival. Positive surgical margins (PSM) are cancer cells that stay at the edge of the resection specimen and have negative prognostic consequences across diverse tumour types, necessitating further (adjuvant) treatments that involve considerable expense and inconvenience for the patient and the healthcare system [3]. Oral cancers have the highest prevalence of PSM. Among sex-specific cancers, ovarian and prostate cancers have the highest prevalence in women and men, respectively [4].

Current techniques for evaluating margin statuses, such as frozen section, imprint cytology, and intraoperative ultrasound, are very useful tools for precision surgery but expensive in terms of time and costs and not always available to surgeons. Furthermore, not all hospitals have experts in the use of this instrumentation applied to tumours [5, 6]. However, there is a clinical need for safe and accurate approaches to defining tumour margins intraoperatively [7]. Molecular image-guided surgery enables the differentiation of tumour and nearby normal tissue, which improves resection accuracy and may reduce PSMs. Along with achieving acceptable margins during tumour removal, this technology can improve the efficiency of debulking surgery and establish new tactics for time-consuming procedures such as sentinel node mapping. It can visualize distinct anatomic structures to protect healthy tissues from harm. Intriguingly, nerve detection still necessitates the development of specific imaging probes, which is currently a priority research topic [8].

Cancer research has thus focused on developing molecular imaging probes to reveal molecular phenotype or even genotype of malignant and normal tissues during image-guided surgery [3, 9–11]. The advancement of molecular pathology understanding has uncovered a plethora of opportunities for image-guided surgery. Targeting cancerous cells based on specific markers not only drives new precision medicine trials, but also enables the development of new molecular imaging procedures [11]. For instance, labelling therapeutic antibodies to translate therapeutic biologicals as imaging agents has already been demonstrated in ongoing surgical trials [12–14].

Advances in nanomedicine have aided in the development of multifunctional and multimodal nanoparticles (NPs). NPs have a substantial impact on cancer imaging because they enable the generation of multimodal contrast agents and surface functionalization for precise molecular recognition [15, 16]. In recent years, several studies investigating the use of nanotechnology platforms for intraoperative imaging have advanced to the clinical stage [17]. Here, we outline the fundamental intraoperative imaging modalities and the most promising contrast agents developed through nanotechnology. In the context of solid tumours, the increased permeability and retention (EPR) effect has proved to be a key factor in cancer nanomedicine design and is a crucial component of drug delivery that targets specific tumours. Active targeting can be employed in addition to EPR-based passive targeting to enhance tumour accumulation and retention in nanomedicine.

## Intraoperative imaging modalities to improve surgical precision

**Fig. 1:**
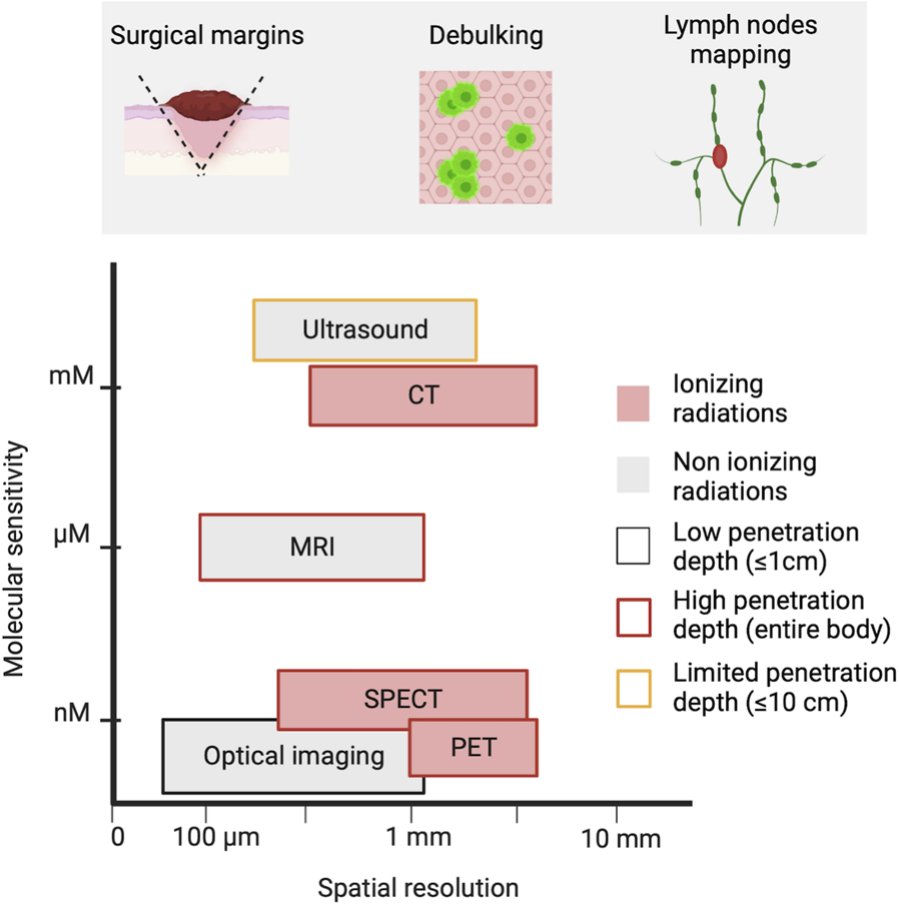
Intraoperative imaging modalities in cancer surgery. Image-guided surgery's potential oncological applications are depicted schematically. The graph shows the sensitivity and spatial resolution ranges of the different imaging modalities

There are numerous medical imaging modalities accessible, including optical imaging, ultrasound, computed tomography (CT), magnetic resonance imaging (MRI), and nuclear medicine imaging, including positron emission tomography (PET) and single-photon emission computerized tomography (SPECT). Each imaging modality has its sensitivity, resolution, and quantitative capabilities, as well as its own set of benefits and inherent restrictions (Fig. 1). The integration of multiple intraoperative imaging techniques has the potential to overcome the limitations of a single imaging modality and represents, therefore, a new task for present and future image-guided surgery research [18]. Novel imaging approaches for intraoperative margin assessment in surgical oncology are rapidly evolving. Heidkamp and colleagues have published a thorough evaluation of intraoperative margin assessment imaging techniques in surgical oncology, as well as data on their technical features, operational feasibility, and diagnostic accuracy [19]. Radiography and ultrasound, in particular, had the highest number of IDEAL 3 and 4 studies, primarily for usage in breast cancer. The most prevalent technique in research investigations was intraoperative fluorescence; however, these only involved IDEAL stages 1 and 2.

### Optical imaging

**Fig. 2:**
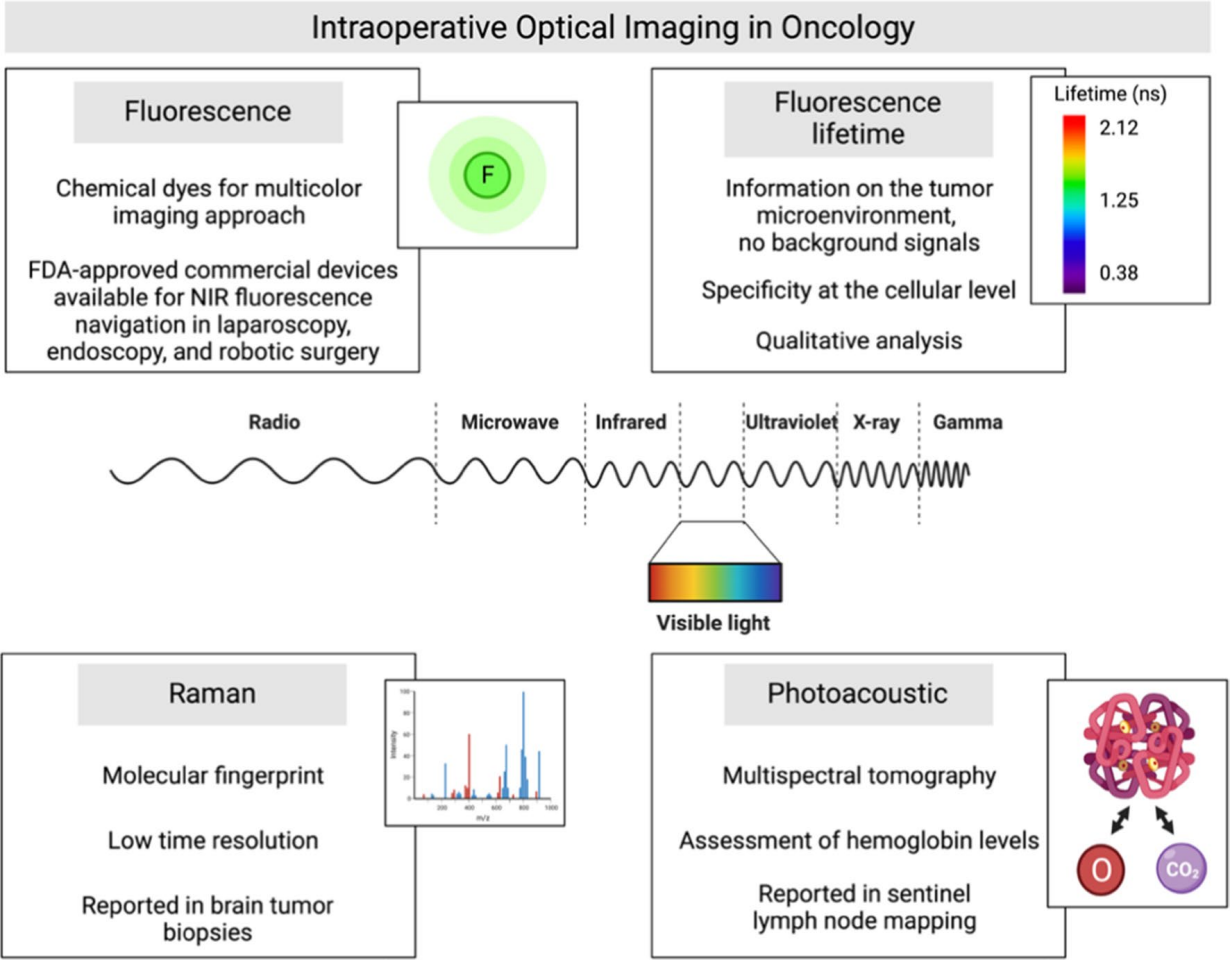
Intraoperative optical imaging in oncology. Optical imaging technologies provide non-invasive visualization, characterization, and often quantification of structures and biological processes at the cellular and molecular levels in therapeutic settings. All imaging modalities are based on the excitation of the tissue by an external light source

Optical imaging technologies rely on non-ionizing radiation to visualize structural, functional and molecular information based on their specific photon absorption and emission or scattering profile. Great efforts have been dedicated to developing an optical imaging system integrated into traditional open-air and minimally invasive surgery. Optical image-guided surgery is an emerging technology that requires the development of contrast agents and dedicated intraoperative camera systems. Over the past few years, substantial progress has been made in both fields [20]. The commonly used optical imaging methods include fluorescence, lifetime fluorescence, photoacoustic imaging and Raman spectroscopy [21, 22] (Fig. 2).

#### Fluorescence imaging

Near-infrared (NIR) fluorescence modalities are the optical imaging approaches chosen for intraoperative image-guided surgery [10, 11, 23]. In vivo imaging in the NIR range (700–900 nm) is superior to that in the visible spectrum due to its low scattering, diminished tissue autofluorescence, and relatively high tissue penetration [24]. The first FDA-approved NIR dye, indocyanine green (ICG), is the most commonly used fluorophore and has shown promising results in image-guided surgery for many organs. Utility for functional imaging in different domains such as identifying lymphatic structures, tumour localization, angiography of blood supply of various organs, thoracic duct visualization, tracheal blood flow analysis, and sentinel lymph node (SLN) biopsy has dominated ICG literature in the field of surgical oncology [25]. The intrinsic limitations of ICG are its short half-life and the lack of a tumour-specific interaction mechanism as a passive fluorescent dye.

There are many commercial devices, FDA-approved, available for NIR fluorescence navigation in laparoscopy, endoscopy, and robotic surgery; an example is the firefly camera integration in the da Vinci Robotic platform [26]. Of interest, there has recently been reported a medical imaging projection system (MIPS) that provides a continuous real-time projection of ICG fluorescence images directly on the surgical field and overcomes the problem of recurrent dimming of operating room lights [27].

Fluorescence can safely be used in several oncology contexts, such as (i) in advanced epithelial ovarian cancer to evaluate invisible microscopic peritoneal metastasis with a high negative predictive value [28]; (ii) for multiple indications in neuro-oncology for resection to the functional limit of the peritumoral region with a greater extent of resection and better outcomes [29, 30]; (iii) to identify structures, including the prostate, neurovascular bundle and lymph nodes in robot-assisted radical prostatectomy [31]; (iv) to better define the borders of the surgical resection in head and neck cancer [13, 32]; (v) for sentinel lymph node mapping in early-stage cervical and endometrial cancer [33]; (vi) to support hepatic resection in liver surgery [34]. This surgical approach improves the current ablation technology, showing promising potential for the complete treatment of more prominent or irregular malignant lesions in the liver and other solid organs.

Notwithstanding attractive and intriguing properties, there are drawbacks of fluorescent imaging that include significant attenuation as the signal travels more in-depth in the tissue and interference from white surgical light during open surgery. Fluorescent imaging of tumour lesions can be challenging where contrast agent uptake is not specific. In an ideal condition, a minimum signal-to-background ratio of 1.5 is required to discriminate fluorescent lesions and guide surgical decision-making [35]. Because of this potential limitation, the development of tumour-targeting probes for fluorescence-guided surgery can improve the chance of achieving complete resection of tumours with negative surgical margins and identification of occult foci of disease.

We are witnessing, on the one hand, a significant advance in the field of fluorescence chemical dye synthesis, with the development of molecules that make possible a multicolour imaging approach [36]; on the other hand, engineering progress has led to the development of new signal detectors capable of encoding multiplicities of signals with efficient spectral separation and sensitivity at low concentrations.

#### Fluorescence lifetime imaging

Fluorescence lifetime techniques have been used for biological and medical imaging as they display sensitivity to the chemical structures of probes and their microenvironment. Previous studies demonstrated that lifetime analysis of exogenous NIR probes effectively provides information about the tumour microenvironment and eliminates background signals, thus improving probes distribution data [37, 38]. Recently, an epidermal growth factor receptor (EGFR) targeted NIR fluorescent probe enabled a fluorescence lifetime imaging approach to enhance tumour contrast with a proven safety profile in humans, suggesting a strong potential for clinical applications in image-guided surgery, cancer diagnostics and staging [39].

#### Raman spectroscopy

Raman spectroscopy can accurately differentiate between tumours and healthy tissue according to their molecular composition [40]. Raman imaging can provide an intrinsic “molecular fingerprint” of the tissue by analysing molecule vibrations (referred to as Raman scattering). Raman techniques offer label-free molecular contrast, albeit with a low signal-to-noise ratio, due to the weak Raman scattering cross-sections. In Raman spectroscopy, despite the advantage of high specificity, the signal intensity is low, and imaging measurements are primarily performed punctually on microscopic tissue volumes, making whole-field assessment temporally inaccessible [40]. Time resolution may have hampered the Raman imaging clinical implementation, and the technological advancement in this field is focused on enhancement of signal and speed acquisition. The entrance of machine learning and deep learning models in the data analysis has contributed to overcoming some of the method’s limits [41].

Raman spectroscopy application for image-guided cancer surgery has recently been explored in brain tumour biopsies, where stimulated Raman scattering microscopy allowed to image and detect tumours infiltrating the human brain [42]. Stimulated Raman histology has enabled rapid diagnostic histology data during pediatric brain tumour surgery, thus improving decision-making [43]. Jermyn et al. adapted Raman spectroscopy for the operating room by developing an imaging technique that uses a commercially available, handheld contact probe technique for live, local detection of cancer cells in the human brain with a sensitivity of 93% and a specificity of 91% [44]. At a preclinical level, Raman spectroscopy demonstrated its potential for peripheral nerve visualisation and identification and for discriminating vital structures during oncological surgery [45, 46].

#### Photoacoustic imaging

Photoacoustic imaging is a clinically emerging modality for molecular imaging of cancer, enabling affordable, real-time non-invasive assessment of tissue oxyhemoglobin (HbO2) and deoxyhemoglobin (Hb) with optical contrast at a high Spatio-temporal resolution [47]. This approach relies on the excitation of short-pulsed laser light in the NIR range to induce the photoacoustic effect in targeted tissues, which results in detectable ultrasound waves generated by thermoelastic expansion [48]. Several studies have investigated the potential of photoacoustic imaging to guide various interventions such as drug delivery, treatment planning and evaluation, surgeries, and biopsies [49]. The multispectral optoacoustic tomography (MSOT) potential combined with ICG has been shown for SLN mapping and is a promising approach to reducing the number of patients subjected to SLN surgical excision by “ruling out” metastasis [50]. Vonk and colleagues report on employing MSOT for in vivo imaging of neck lymph nodes in oral cancer patients using cetuximab-800CW, a tumour-specific fluorescent tracer [51]. MSOT-based assessment of haemoglobin levels can be used as a surrogate of inflammation, and at the intestinal wall, it allows to distinguish the active disease from remission in patients with Crohn’s disease [52].

Recently, US and optoacoustic tomography fusion has demonstrated the ability to decode the distribution of lipids and collagen, aiming at differentiating benign and malignant lesions on the breast [53]. This holds great potential in an intra-operative setting to evaluate tumour margins.

### Radio-guided surgery

**Fig. 3:**
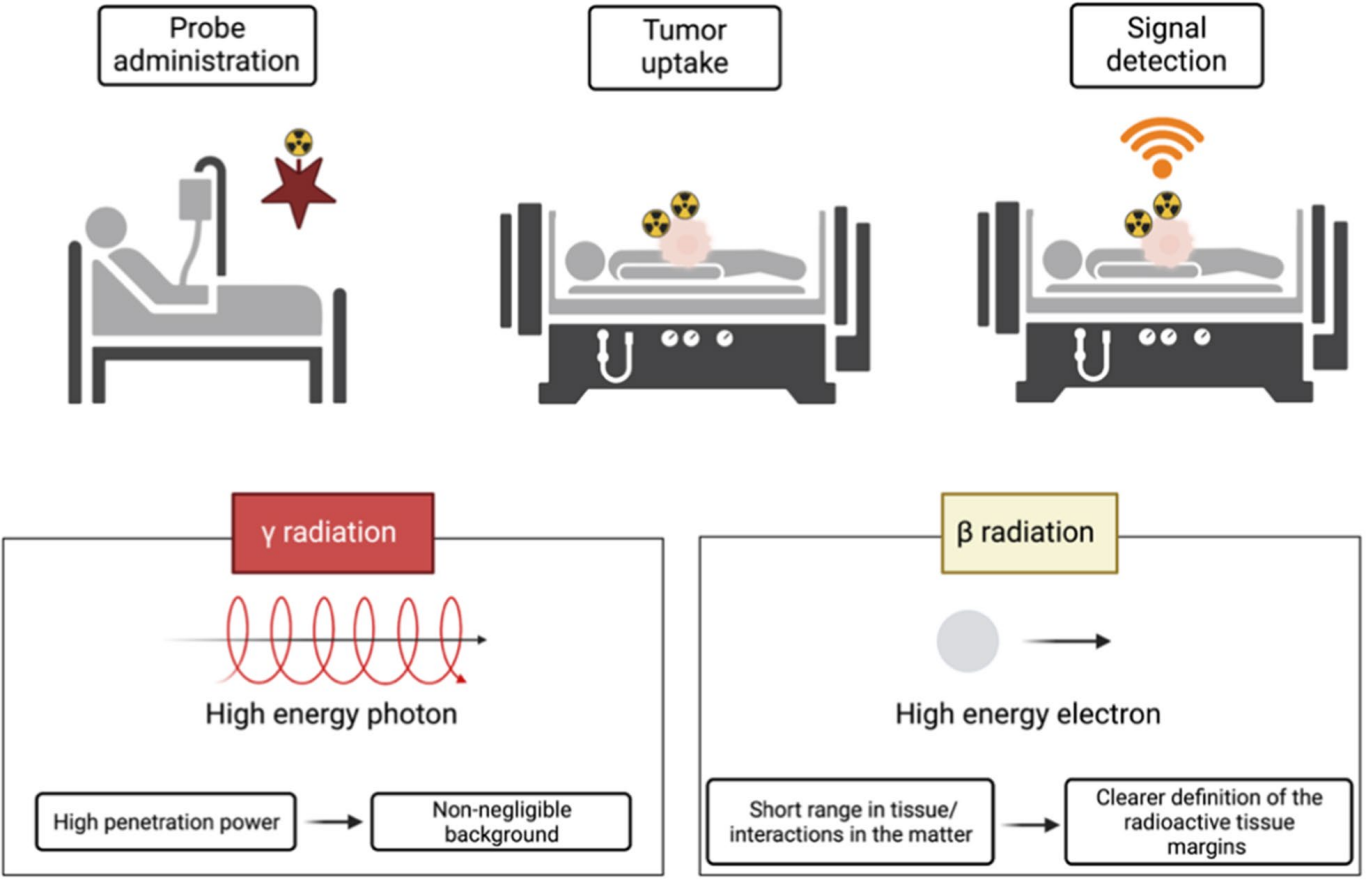
Schematic illustration of a typical radio-guided surgical procedure. Stages of the procedure include: (1) a radio-labelled tracer administered to the patient before the operation; (2) the radiopharmaceutical mainly absorbed by the tumour; (3) after the tumour mass removal, the surgeon scans the lesion with a radiation detection probe and searches for targeted tumour residues in real time. The lower images show the effect of the proposed procedure with γ-emitter tracers, and with β-emitting tracers

Radio-guided surgery (RGS) is a surgical technique developed about 60 years ago that is crucial for those tumours for which resection is the only possible therapy [54]. It allows the surgeon to assess the completeness of the tumour lesion resection in realtime, minimizing the amount of healthy tissue removed [54]. The RGS consists of complex procedures which, using a suitable probe for the detection of radioactivity in the operative field, allows the identification and, therefore, the surgical excision of a tissue that has been “marked” presurgical with a specific radiopharmaceutical (Fig. 3) [55]. Radiopharmaceuticals are biologically active molecules labelled by radionuclides that provide a source of ionizing radiation mainly applied in diagnostic imaging and therapy [56]. The radiopharmaceutical is administered to the patient before surgery, temporarily making the body a source of radiation to detect with a specific instrument, an intraoperative probe sensitive to the emissions released by the drug [55]. The radiopharmaceutical is administered to the patient and absorbed by the target tissue. Therefore, during surgery, the surgeon can position the probe in correspondence with the lesion since the probe detects the emissions released by tissue-enhancing in real time [55].

The RGS probe converts the radioactivity detected intraoperatively into a numerical signal and into an acoustic signal of intensity and frequency proportional to the activity in the region under examination. The “ideal” probe for RGS should have the best sensitivity, spatial resolution, and energy resolution [57]. Unfortunately, these three parameters cannot be optimized simultaneously, considering that sensitivity and spatial resolution are inversely correlated. It is, therefore, often necessary to reach the best possible “trade-off” between sensitivity and spatial resolution, a trade-off that can occur at different levels for the different applications of RGS. Numerous intraoperative probe systems have been developed and then marketed for use in radio-guided surgery, but we can classify them into two general categories, based on the specific type of radiation detected: gamma probes and gamma radiation detection systems, as well as beta probes and beta radiation detection systems [55]. The type of probe to be used during the surgery depends on the type of tumour, the characteristics of the radiopharmaceutical, and the preferences of the oncologist and surgeon. The intraoperative device detects the specific activity in the radiopharmaceutical uptake area and translates the intensity of the radioactivity recorded into a digital value [55]. This number is expressed in counts per second (cps) and in an acoustic signal of intensity and frequency directly proportional to the detected activity. The acoustic signal emitted by the probe represents a guide for the surgeon to identify the lesion and any other residues present after the surgical removal of the tumour. The standardized number produced by the probe is interpreted as the relationship between the signal and the background (Tumor-to-Background Activity Ratio, TBR), that is, between the activity originating from the lesion that picked up the radiopharmaceutical administered to the patient and the activity originating from the area adjacent to the tumour, radiation originating from non-diseased absorbing tissue, caused by electromagnetic radiation interactions with matter.

RGS represents, for the surgeon, an additional tool, providing intraoperative information from a radiation detection device in the form of acoustic signals of intensity and/or frequency proportional to the amount of radiation detected for the sole purpose of guiding the successful performance of the surgical procedure.

#### Gamma probe

The first RGS description involving a gamma-sensing system, was reported in 1956 at the Oak Ridge Institute of Nuclear Studies Medical Hospital. On that occasion, the authors of the study published the results obtained on a patient with thyroid cancer, for whom, after the administration of a gamma radiation emitter (I^131^), the area of the thyroid tissue was successfully localized [58]. The most important clinical applications of gamma detection technology are sentinel lymph node research and the Radioguided Occult Lesion Localization (ROLL) technique [54, 59]. The gamma radiation high-penetration capacity is the only authentic limit for this type of probe. In fact, γ-rays cross the human body effortlessly and for large quantities of tissue (it is necessary to stop thicknesses of a few centimeters of lead or decimetres of concrete), and the possible absorption of the tracer in the area of healthy tissue near the lesion represents a non-negligible background [54]. It can sometimes prevent the applicability of the γ-RGS technique, so to overcome the problems encountered with gamma probes, new particles have been sought, and innovative probe prototypes have been proposed.

#### Beta probe

As for γ-RGS, it is clear that most of its drawbacks arise from the photons use as decay particles. The development of probes designed to detect beta particles (β+ and β−) is one of the most recent developments in the field of RGS [55]. Since β radiation (positrons or electrons) has a short range in tissue, these probes are ideal for detecting tracers in tumours at the surface of the surgical field. The first option is to employ a radioactive atom that decays by emitting positrons (β+ particles). Several β+ emitting isotopes are already commonly used in nuclear medicine. The most common one is the ^18^F, utilized in ^18^F-FDG PET exams. Another advantage of this approach is related to the interactions of positrons in the matter. Positrons (being charged particles) interact much more than photons. Therefore, their range is reduced to a few millimetres for β+ particles of about 1 MeV. Due to their nature as antiparticles, they annihilate encountering electrons in the traversed tissue, producing a couple of back-to-back photons of 511 keV in energy. The low penetration range results in the double advantage of a narrow path within the body, assuring better spatial resolution than photons, thinner detectors, and more miniature probes. Consequently, the β+ emitters solve some of the main limitations of γ-RGS, such as long gamma radiation penetration and the resulting loss of spatial resolution.

However, some problems remain with this strategy, primarily due to physical causes such as the annihilation photon background [55, 60, 61]. The third approach in RGS, which aims to overcome all the limitations outlined thus far, would be represented by β− radiation. The search for possible particle emitters was prompted by difficulties encountered during radio-guided surgery with β+ probes. The β− particles penetrate a few millimetres of tissue. They have energy distributed in a continuous spectrum extending between 0 and the incident β− radiation, which can be ignored because it is transferred to the photons produced rather than the medium.

The β− emitting radiopharmaceuticals are less sensitive to the presence of background radiation. They represent a problem and a source of degradation of the resolution of the probe, and, therefore, this new device can operate with low background detecting the margins of the tumour radioactive-tissue in more detail, with a substantial reduction (due to β− particles low penetration) of the dose given to the medical staff [55, 60]. The detection of β− emission had been proposed at the outset of RGS development. However, it was soon abandoned because of the emission phenomenology and because the available tools and materials did not meet the prerequisites needed. Modern technologies make it possible to develop RGS with these particles and quantify the impact of this innovation in the sector. In recent years, this proposed novel technique of RGS proved not only to be theoretically possible [62] but also its actual feasibility has been demonstrated with ex-vivo tests on meningioma samples marked with ^90^Y-DOTATOC [63].

### Ultrasound

Due to its low cost, lack of reliance on radiation, and noninvasive nature, ultrasound imaging has become widely used in clinical settings. Moreover, ultrasound imaging systems can be portable, which accounts for their widespread use in clinics. Ultrasound contrast agents increase the differential in acoustic impedance across tissues or at vascular/tissue interfaces, amplifying the reflected acoustic echoes. The acceptance criteria for ultrasound contrast agents include excellent acoustic impedance changes, appropriate stability, a proper size that allows extravasation of the vascular space, good compatibility, and the required safety measures for live tissues [64]. The particle size, core material, compounds, and shell thickness can all impact the acoustic echo intensity of ultrasound contrast agents.

Intraoperative contrast-enhanced ultrasound is a sophisticated approach that uses an intravenous microbubble-based contrast agent to provide valuable information about tumour biological properties through direct observation of its vascular pattern [65]. Neuro-oncological surgery is the initial application field of intraoperative ultrasound [66]. During neurosurgical procedures, ultrasound imaging accurately identifies brain tumour lesions and maximizes the resection area while preserving normal brain parenchyma in both intra- and extra-axial lesions [66, 67]. As a result, neurological functions are better preserved, which reduces mortality and increases progression-free survival. Although liver imaging is the most widely used application, recommendations for non-hepatic conditions have also been established [68].

Intraoperative ultrasound imaging is also employed in gynecologic surgery (particularly for ovarian cancer) [69, 70], general surgery for colon cancer [71], and breast cancer [72].

### Magnetic resonance imaging (MRI)

Using MRI to guide surgical navigation has distinct advantages over other imaging modalities because MRI delivers efficient soft-tissue contrast and spatial resolution. Also, MRI allows for simultaneous imaging of soft tissue and interventional equipment (e.g., needles and catheters), allowing the interventional plan to be adjusted and controlled interactively. Furthermore, as a multiparametric imaging approach, MRI can detect several physiological functions such as blood oxygen level, flow, and temperature [73].

MRI is the gold standard for diagnostic imaging in neurosurgery and is employed in neuro-oncology for pre-surgical planning, surgical navigation, and monitoring therapy response [74]. MR images aid in diagnosing glioma by examining the association between the glioma infiltration area and the eloquent area of the connecting fibres [75]. As a result, intraoperative MRI can help neurosurgeons determine the extent of removal, the distance between the excision site and eloquent regions, and how to rectify brain shifts in the neuro-navigation system [75]. Prostate surgery is one of the most active research fields in MRI-guided robotics. Because of their potential to offer comprehensive anatomical pictures in real-time, MRI-guided robots have been created to provide intraoperative imaging guidance and automatic targeting [73]. MRI-guided imaging finds another substantial application field in robotic system implementation for breast tumour biopsy and therapy.

### Computed tomography (CT)

With a high frequency of usage and hospital availability, CT is one of the most potent tools for guiding diagnosis and treatment [76]. Intraoperative CT was introduced in the early 2000s as a portable device and has been utilized successfully in surgical oncology [77–79]. The resolution of the soft-tissue image is improving as multidetector CT technology advances [77]. However, contrast agents with high X-ray attenuation, often including high atomic number elements such as iodine, barium, and gold, are required to boost the sensitivity of CT scanning and improve imaging of the tissue of interest. Current CT contrast agents, primarily based on small iodinated compounds, have quick elimination and limited efficacy: only massive doses at molar concentrations can generate appropriate contrast for CT imaging [80]. The relevance of CT as one of the fundamental radiological modalities used in biomedical imaging has accelerated the development of NPs as next-generation CT contrast agents [81].

## Nanotechnology approaches for image-guided surgery

The EPR effect has become a significant driver of cancer nanomedicine design in the therapy of solid tumours, serving as a critical cornerstone of tumour-targeted drug delivery [82, 83]. EPR-mediated tumour accumulation has traditionally been thought to be caused by long-circulating NPs with hydrodynamic diameters wider than the renal clearance threshold, which can extravasate from leaky tumour vasculature. However, recent research has looked into ways to broaden the traditional concept of EPR-based tumour targeting [83]. The mechanism by which NPs enter solid tumours was more complex than previously thought, and immune cells in the tumour microenvironment play critical roles in the accumulation, retention, and tumoral distribution of nanomedicines [83, 84].

The fact that the EPR effect is a blood vessel phenomenon that heavily depends on tumour blood flow is a crucial issue that needs to be addressed [85]. Many clinical cancers, particularly advanced late-stage and refractor cancers, are poor in tumour blood flow due to high coagulation activity and thrombi formation, resulting in an unsatisfactory EPR effect. The EPR impact is significantly broader in small animal xenograft tumour models, which are routinely employed in preclinical settings to explore cancer nanomedicines, than in tumours developing in humans. As a result, assessing the degree of EPR effect in tumours using non-invasive imaging is a promising strategy for stratifying patients for cancer nanomedicine treatment.

**Fig. 4:**
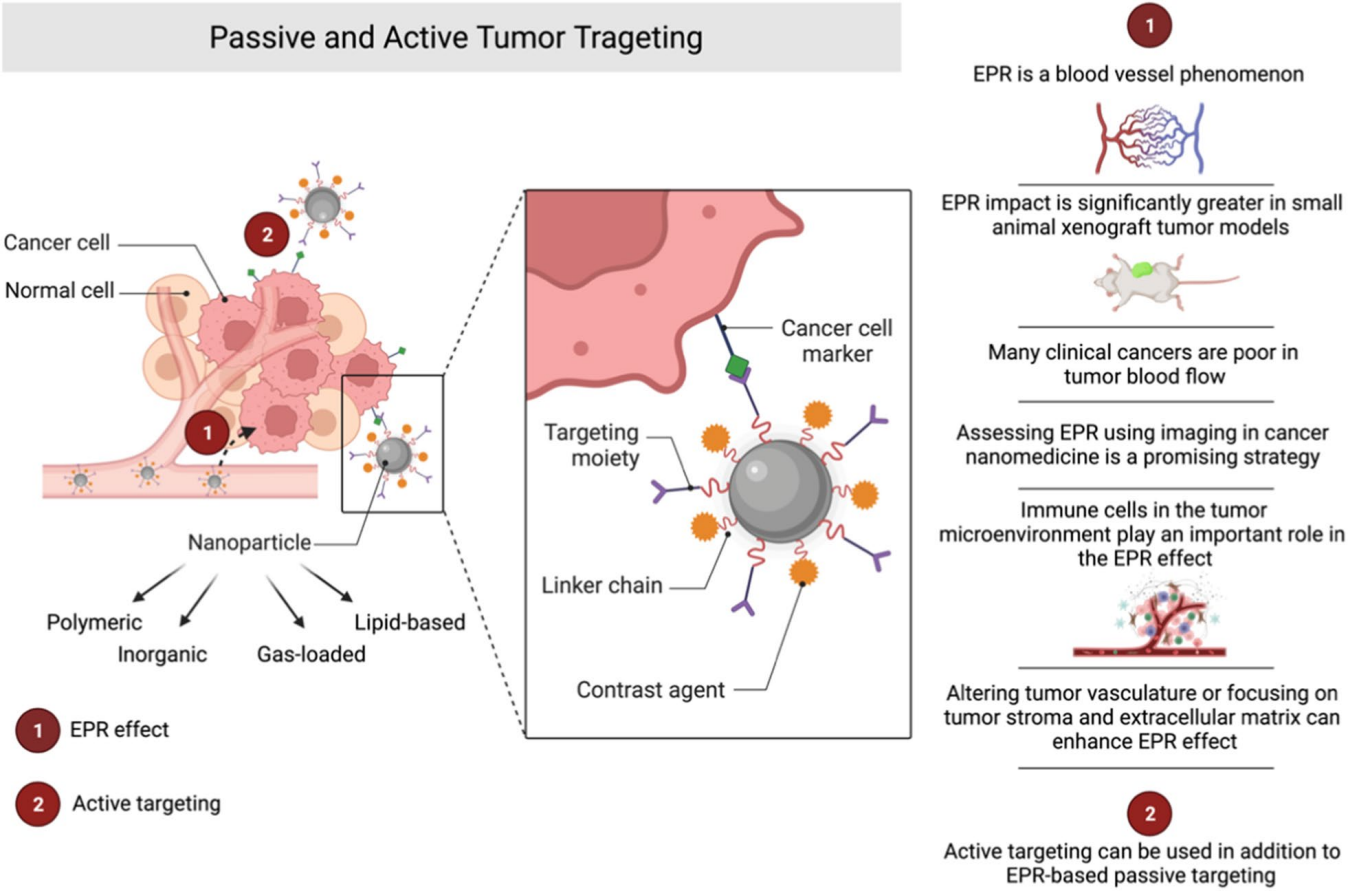
The enhanced permeability and retention (EPR) effect is a critical element in the design of cancer nanomedicine and is an essential part of drug delivery that targets solid tumours. Active targeting added to passive targeting based on EPR can improve tumour accumulation and retention. Passive accumulation occurs initially (1), with target-specific binding arising as a synergic strategy (2), even if the nanocarrier is intended for active targeting

The EPR effect is a dynamic phenomenon that can empowered by altering vascular mediators. Numerous investigated methods successfully enhanced the EPR effect-based nanomedicine therapy, such as the alteration of the tumour vasculature or focusing on tumour stroma and extracellular matrix [85]. Active targeting added to EPR-based passive targeting (Fig. 4) can improve nanomedicine tumour accumulation and retention. Antibodies, antibody fragments (e.g., nanobodies), and peptides are examples of tumour-targeting ligands. Even if the nanocarrier is designed for active targeting, passive accumulation happens first, followed by target-specific binding as a supplementary technique. The possibility of enhanced targeting leads to increased treatment efficacy and fewer adverse effects caused by drug accumulation in healthy tissues. As a result, actively targeted nanotherapeutics have generated considerable interest as a possible method for addressing unmet medical needs [15].

**Fig. 5:**
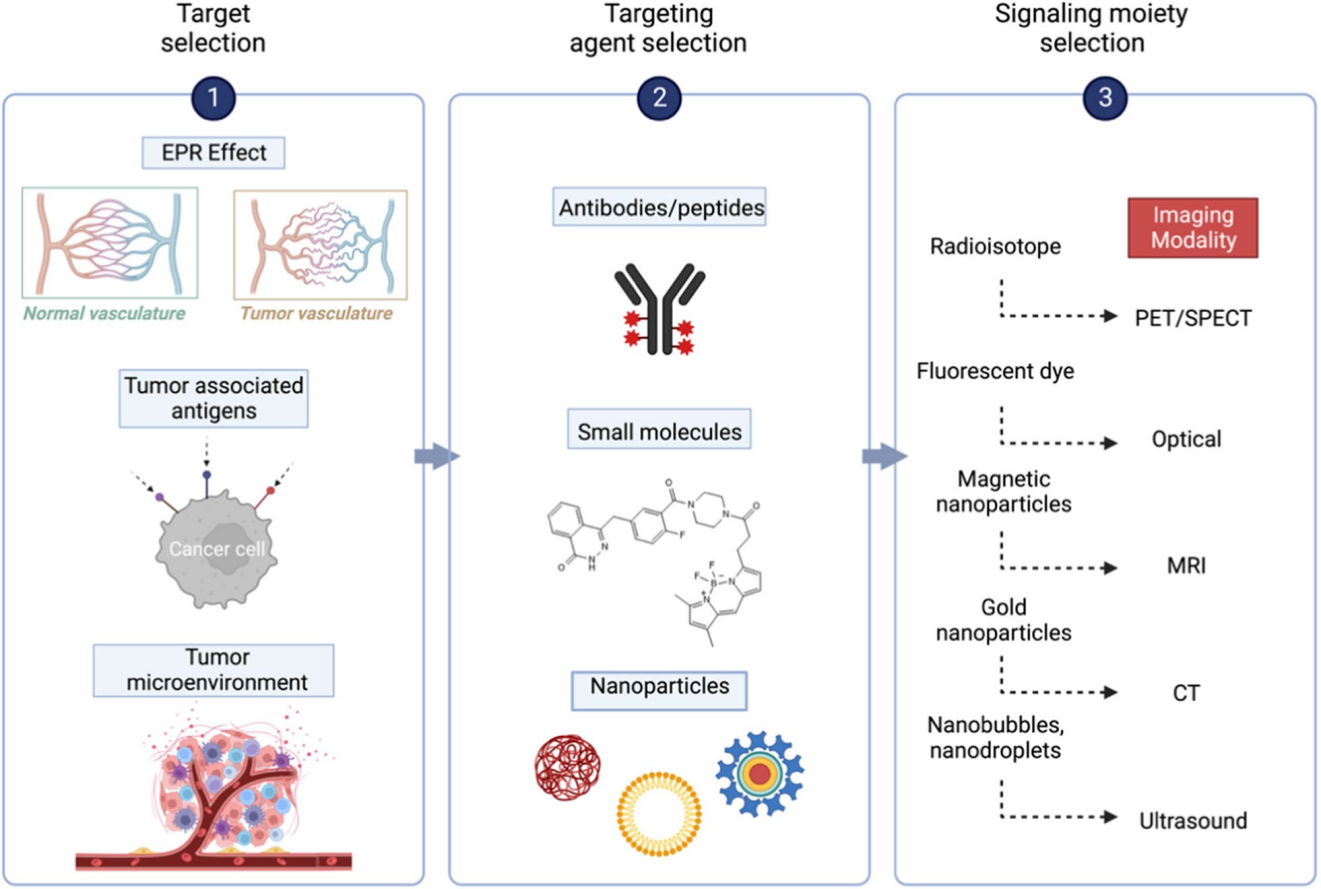
Different stages in the development of novel tumour-targeted imaging agents. (1) Many approaches might be applied to highlight the tissue of interest. (2) The appropriate target selection should be combined with identifying a targeting agent/binder to interact with the target. In general, downsizing the binder shortened the half-life of the imaging agent in circulation, reducing background signal intensity. Synthetic binders, such as small molecules and nanoparticles, are appealing for active targeting because they may be modified using various chemical techniques. (3) The signal moiety generates the signal for imaging applications

Tumour-targeted image-guided surgery development has advanced dramatically, initiating a new field of research centred on real-time intraoperative visualization of cellular processes to improve cancer surgery precision. Imaging-agents can be made to precisely bind to or interact with a target protein associated with a cancer hallmark using several methods. It is possible to identify various stages in the development and clinical translation of new tumour-targeted imaging agents (Fig. 5). First, a viable target is selected based on clinical need. Following that, a targeting agent is created. Finally, the signalling moiety is chosen based on the imaging modality. Following validation, the imaging agent (which consists of the targeted agent and the signalling moiety) is tested in early-stage clinical studies.

**Fig. 6:**
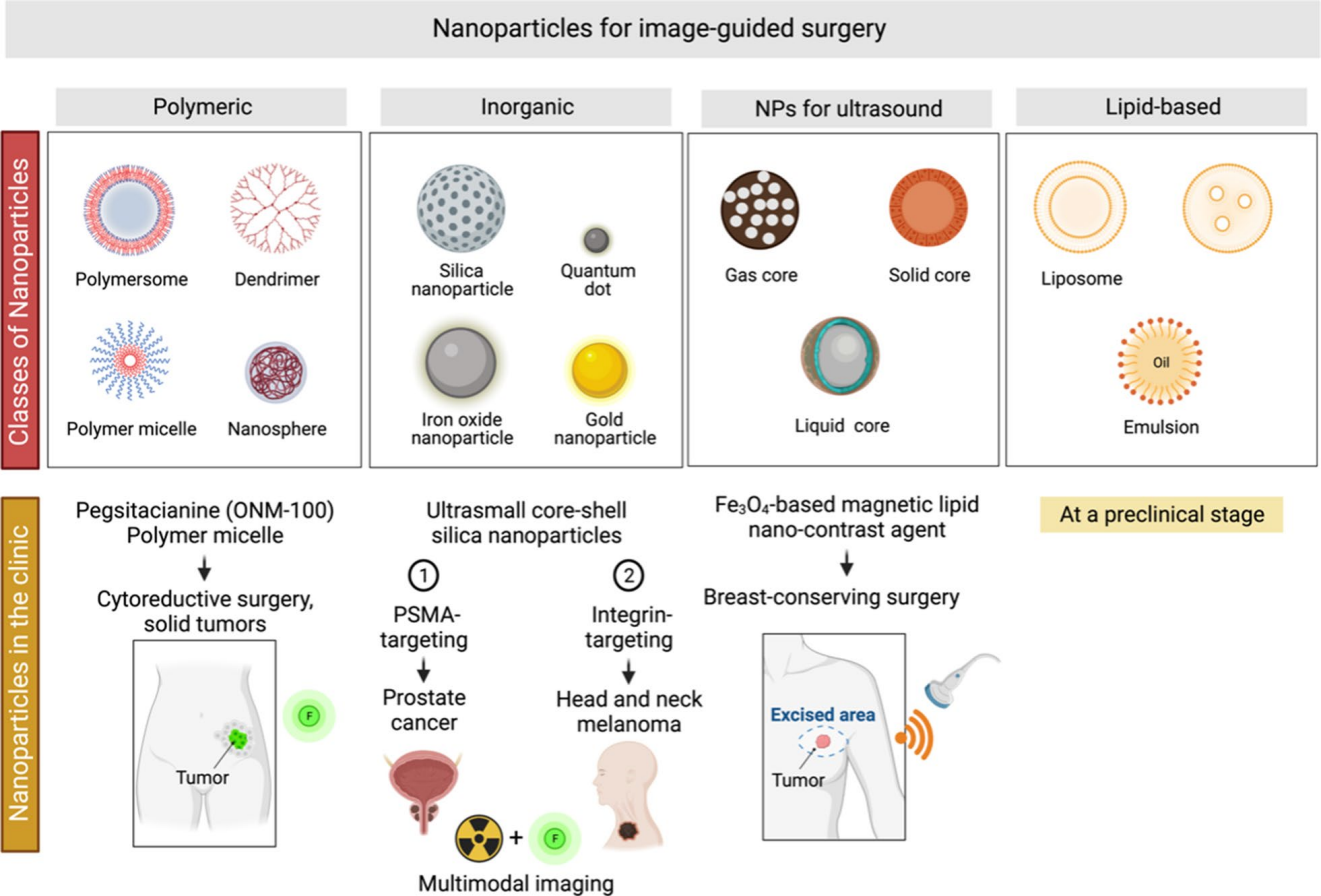
The four main categories of targeting agents based on NPs used in image-guided surgery are polymeric, inorganic, lipid-based, and NPs for the ultrasound. Some NP-based formulations have already been tested in different clinical settings

NPs, which can be categorized into four classes, comprise a large target agent category (Fig. 6). In the context of image-guided surgery, the four primary types of NP-based targeting agents are inorganic, polymeric, lipid-based, and ultrasound.

### Nanoparticles for image-guided surgery in the clinic

Although the development of nanotechnology for image-guided surgery is now mainly in the preclinical stage, there are some intriguing clinical applications in the pipeline (Table 1). In particular, five imaging NP-based formulations were tested in different clinical settings.

**Table 1:** Registered clinical studies using nano-sized particles for image-guided surgery

Study title	Nanoparticle (NP)	ClinicalTrials.gov identifier (phase)	Commentary
Potential Role for Carbon Nanoparticles to Guide Central Neck Dissection in Patients with Papillary Thyroid Cancer	Carbon NPs	NCT02724176	In contrast to the methylene blue approach, carbon nanoparticles (CNs) can adequately predict thyroid microcarcinoma patients' lymph node (LN) status and retain the durability of sentinel LN imaging. The CN technique makes it easier to identify and select patients who would most likely benefit from cervical LN dissection [86]. Colorectal cancer lymph nodes can be more easily found using tracers like activated CNs suspension in vivo [87]
Effect of Carbon nanoparticle suspension injection vs ICG in Lymph Node Tracing During Gastrectomy (FUTURE-01)	Carbon NPs	NCT05229874	With the fluorescence imaging feature of the Da Vinci robot surgery XI system, indocyanine green lymph node tracking navigation technology can be used throughout the procedure. Furthermore, the equipment has no impact on the lymph node tracking effect of the injection of nanocarbon suspension. The Da Vinci robot's flexible mechanical arm and the use of lymph node tracing technology increase the thoroughness of the lymph node dissection during gastric cancer surgery while also making the procedure less challenging. To assist clinical surgeons and provide them with an option, the lymph node tracer approach that is more suited for robot surgical systems is chosen comparing two tracer methods
Carbon Nanoparticles vs Indocyanine Green	Carbon NPs	NCT04759820	While both carbon NPs and ICG as lymph node tracers in colorectal cancer surgeries have their value, the study aims to compare two tracers to determine the most effective. It will help to further improve postoperative lymph node inspection, precise postoperative adjuvant therapy, and patient long-term survival
Application of Carbon Nanoparticles in Laparoscopic Colorectal Surgery	Carbon NPs	NCT03350945	The project aims to assess the application of carbon NPs for tumour localization and lymph node mapping during laparoscopic colorectal surgery in terms of its security, efficacy, accuracy, and cost-effectiveness
The Use of Nanoparticles to Guide the Surgical Treatment of Prostate Cancer	64Cu-NOTA-PSMA-PEG-Cy5.5-C'	NCT04167969	This study examines the safety of identifying tumour cells before and during prostate cancer surgery utilizing the 64Cu-NOTA-PSMA-PEG-Cy5.5-C' dot tracer. The study aims to determine whether PET/MRI scans performed after the injection of this experimental tracer are more accurate than standard imaging scans for locating prostate tumour cell masses. The tracer's biodistribution will be monitored, and the use of this tracer in patients undergoing surgery for prostate cancer will be studied for the first time [88]
Targeted Silica Nanoparticles for Real-Time Image-Guided Intraoperative Mapping of Nodal Metastases	cRGDY-PEG-Cy5.5	NCT02106598	Patients received standard-of-care technetium Tc 99 m sulfur colloid before receiving an intradermal microdose of integrin-targeting, dye-encapsulated NPs with polyethylene glycol chains on their surfaces. These NPs are called cRGDY-PEG-Cy5.5. This study established the viability and safety of fluorescence-guided sentinel LN biopsy using cRGDY-PEG-Cy5.5 in head and neck melanoma. With the potential to reduce procedural risks, this method holds promise for enhancing lymphatic mapping and SLN biopsy operations [89]
A Study to Evaluate Diagnostic Performance and Safety of Pegsitacianine, an Intraoperative Fluorescence Imaging Agent for the Detection of Lung Malignancies in Patients Undergoing Routine Surgery	Pegsitacianine (ONM-100)	NCT05048082	A Phase 2, Single-Dose, Open-Label Study to Assess the Diagnostic Performance and Safety of Pegsitacianine, an Intraoperative Fluorescence Imaging Agent for Lung Malignancies Detection of in Patients Having Routine Surgery. Micelles covalently coupled to indocyanine green constitute the NP-based fluorescent imaging agent pegsitacianine. Other Names: ONM-100
A Study to Evaluate Pegsitacianine, an Intraoperative Fluorescence Imaging Agent for the Detection of Peritoneal Metastases in Patients Undergoing Cytoreductive Surgery	Pegsitacianine (ONM-100)	NCT04950166	Patients undergoing cytoreductive surgery are given pegsitacianine to assist surgeons in identifying peritoneal metastases. Pegsitacianine takes advantage of the constant pH variations between healthy and malignant tissues. The localization within the tumour microenvironment offers a highly-sensitive, focused fluorescence response, enabling the detection of primary tumours, their margins, metastatic disease, and lymph nodes containing tumours
A Study to Evaluate ONM-100, an Intraoperative Fluorescence Imaging Agent for the Detection of Cancer	Pegsitacianine (ONM-100)	NCT03735680	The purpose of this study is to assess the diagnostic performance, safety, and timeliness of ONM-100 post-dose imaging, in patients with solid tumours undergoing standard surgery (breast cancer, head and neck squamous cell carcinoma, colorectal cancer, prostate cancer, ovarian cancer, urothelial carcinoma, non-small cell lung cancer)
Cathepsin Activatable Fluorescent Probe	LUM015	NCT01626066	The goal of this study is to establish a safe dosage of LUM015. According to a mouse-human phase 1 co-clinical study, the tumour selectivity of protease-activated imaging probes, such as LUM015, relays on both biodistribution and enzyme activity. LUM015 is selectively delivered to tumours, where proteases then activate it [90]
Feasibility of the LUM Imaging System for Detection of Gastrointestinal Cancers	LUM015	NCT02584244	This feasibility study's main objective is to evaluate the LUM015's initial safety and effectiveness for ex vivo far-red imaging of colorectal, pancreatic, and oesophageal malignancies (adenocarcinoma). LUM015 will be given 2–6 h before tumour excision colorectal and oesophageal cancer cases. For pancreatic patients, the injection will take place one hour before the scheduled start of the surgery
Feasibility Study of Intraoperative Imaging in Breast Cancer	LUM015	NCT02438358	During breast cancer surgery, the LUM015/LUM2.6 Imaging System enables the real-time detection of residual tumours in the lumpectomy cavity and may lower the percentage of positive margins while also lowering the amount of tissue removed [91]
Feasibility Study of Intraoperative Detection of Residual Cancer in Breast Cancer Patients	LUM015	NCT04440982	Enrollment and interim analysis are completed for Cohort 1. The fluorescence signals obtained, were within the predicted range, and no additional concerns, particular to this patient cohort, were found. Cohort 2 now has four more patients [92]
Feasibility of the LUM Imaging System for Detection of Prostate Cancer	LUM015	NCT03441464	This feasibility study's primary goal is to assess whether administering LUM015 to patients having radical prostatectomy for prostate cancer would cause tumor tissue from ex vivo specimens to fluoresce positively. Imaging and analyses of both tumor and healthy tissue will be performed
Feasibility of the LUM Imaging System for Detection of Cancer in the Brain	LUM015	NCT03717142	This study aims to evaluate the LUM Imaging System's safety and efficacy in imaging primary and metastatic cancer in the brain. This involves determining a starting dose of LUM015 for molecular imaging of low-grade gliomas, glioblastomas, and cancer masses that have metastasized to the brain
Feasibility of the LUM Imaging System for Peritoneal Surface Malignancies	LUM015	NCT03834272	This single-site feasibility research will evaluate the LUM Imaging System’s initial safety and effectiveness for in vivo imaging of metastases to the peritoneum from primary gastrointestinal cancer, ovarian cancer, and mesothelioma. It feasibility study is divided into two parts: (a) a dosage escalation phase to determine the appropriate dose, followed by (b) further patient enrolment to develop the tumor detection algorithm
Intraoperative Detection of Residual Cancer in Breast Cancer	LUM015	NCT03321929	It is a multi-site, non-randomized, open-label trial to collect data on the safety and efficacy of the LUM Imaging System in finding residual cancer in breast cancer patients. LUM05's safety profile was comparable to that of other imaging agents used in breast cancer surgery, and it was related to a lower need for a second surgery in patients who received intraoperative excision of LUM05-guided shaves [93]
Investigation of Novel Surgical Imaging for Tumor Excision (INSITE)	LUM015	NCT03686215	This pivotal study is multi-centre, two-arm, randomized, and blinded. Its goal is to show the safety and effectiveness of the LUM Imaging System in identifying residual cancer in lumpectomy surgery to support surgeons in decreasing the rates of positive margins
Feasibility Study of LUM Imaging System for Pancreatic Cancer	LUM015	NCT04276909	This single-site, non-randomized, open-label trial aims to evaluate the initial safety and effectiveness of the LUM Imaging System for primary pancreatic cancer and primary pancreatic cancer-related peritoneal invasion during surgery. The algorithm for tumour identification will be developed for this indication in this feasibility study

A carbon NP suspension injection contains nanosized carbon particles with an average diameter of 150 nm [86]. When nanocarbon particles are injected into the tissues around the tumour, macrophages quickly engulf them. After entering lymphatic vessels, the particles accumulate in the lymph nodes and stain them black. This approach has made the vital staining of lymph nodes that drain tumours easier. Four documented clinical studies investigated patients with gastric cancer, colorectal cancer, and papillary thyroid carcinoma. Long-term gastric cancer survival depends on complete perigastric lymphadenectomy. It is essential for precise staging of malignancies, selecting the subsequent treatment plan, and improving prognosis [87]. Therefore, developing technologies that can make performing this procedure safer and increase the precision of lymph node dissection is a topic of significant interest. Carbon NPs outperformed methylene blue concerning lymph node staining and positive rate for SLN metastatic disease in thyroid cancer surgery [88]. Other dyes, such as methylene blue and indocyanine green, have been employed to enhance the results of thyroid surgery. The main disadvantage of methylene blue is that it stains the lymph nodes and the parathyroid glands [89], causing intraoperative complications.

A recent Phase I clinical trial involved 10 patients for laparoscopic radical prostatectomy and bilateral pelvic LN dissection or salvage lymph node dissection (NCT04167969). Patients received PSMA-targeting NP injections up to 48 hours before surgery. Chen et al. describe the study’s findings of ultrasmall (sub-8 nm diameter) PSMA-targeting core-shell silica NPs (Cornell prime dots) for dual-modality imaging in pre-operative (PET) and/or intraoperative (optical) settings. The PSMA-targeting platform prevents undesirable accumulations in the salivary glands, kidneys, and reticuloendothelial system while demonstrating bulk renal clearance [90].

Zanoni and colleagues created and tested the ultrasmall integrin-targeting, ultrabright, fluorescent core-shell silica NPs (NCT02106598) [91]. The enhanced brightness and photostability of fluorescent NPs over free dyes are due to the encapsulation of Cy5.5 within the silica core matrix. Furthermore, because αv integrins are known to be overexpressed on the surface of neoangiogenic endothelial and melanoma cells, the particle surface was functionalized with multiple integrin-targeting peptides (i.e., cyclic arginine-glycine-aspartic acid-tyrosine [cRGD]) via polyethylene glycol chains (PEGs) to create cRGDY-PEG-Cy5.5-NPs. As cRGDY-PEG-Cy5.5-NPs were used in procedures where SLNs could be detected and removed, the length of the surgery was estimated to be reduced by 30–50% when compared to technetium Tc 99m sulfur colloid alone. This range represented differences in procedure type, node depth from the skin's surface, the need for nerve dissection, and other technical aspects such as equipment setup.

A Phase I study (Netherlands National Trial Register #7085) explored ONM-100, a molecular imaging probe based on an ultra-pH sensitive amphiphilic polymer coupled with indocyanine green. This tool quickly and irreversibly dissociates to emit fluorescence in the acidic extracellular tumour microenvironment via macromolecular cooperativity at the nanoscale [92]. ONM-100 has also been studied in two more Phase II clinical trials in patients undergoing surgery for peritoneal carcinomatosis (NCT04950166), lung cancer (NCT05048082) and various solid tumours (NCT03735680).

The LUM015 probe, which has a polymer therapeutic chemical configuration, warrants a separate consideration when examining nanotechnology approaches for tumour-targeted imaging. Polymer therapeutics—a broad category that includes polymeric drugs, polymer-drug conjugates, polymer-protein conjugates, polymeric micelles, and polymeric non-viral vectors for gene delivery—are nano-sized medicines [93]. The rationale behind this chemical strategy relies on the ability of polymer conjugation to change a low molecular weight drug's biodistribution. The increased drug molecular weight results in pharmacokinetics substantial alteration at whole-body and cellular levels, making the covalent attachment of the drug to a polymeric carrier particularly appealing. LUM015 is a PEGylated protease-activated fluorescent imaging probe [94]. LUM015 comprises 20-kD polyethylene glycol (PEG) and a Cy5 fluorophore connected to a commercially available fluorescence quencher molecule (QSY21) through a GGRK peptide. Although LUM015 is optically inactive, cathepsins K, L, and S (and, to a lesser extent, B) cleave it to release the quencher, which liberates the optically active fragment. Co-clinical investigations indicate that both biodistribution and enzyme activity is critical for the tumour selectivity of this protease-triggered imaging probe [94]. The tumour/normal ratio is not entirely explained by protease activation; instead, tumour-selective accumulation via the EPR effect is established, supporting the function of PEGylation.

### Polymeric nanoparticles

Polymeric NPs represent nanocarriers unique in terms of nanometric size range from 1 to 1000 nm, high surface area-to-volume ratio, and suitable characteristics for drug release and tumour targeting. The “polymeric NPs” label encompasses nanocapsules as well as nanospheres differing in morphological structure mostly, and the first model can be loaded with drugs or molecules adsorbed on the surface of the polymeric core, whereas in the second the molecules are entrapped in the polymeric network of NPs [95]. In NP formulations, the polymer used can be either of natural or synthetic origin such as saturated poly(-hydroxy esters) [e.g. poly (lactic acid) (PLA), poly (glycolic acid) (PGA), and poly (lactic-co-glycolide) (PLGA)] approved by the FDA. These kinds of polymers are characterized by a high safety profile and biocompatibility, with low levels of immunogenicity and toxicity, and they are completely biodegradable [96]. These properties make polymeric NPs well-suited to promote the accumulation of drugs in the target tissue and improve the treatment of several diseases [97–99].

Polymeric NPs are considered soft NPs that can hold a various cargos, such as fluorophores, therapeutic agents, and inorganic NPs to produce hybrid NPs. Because of the plasticity of soft NPs, many fluorescent compounds can be enclosed, with the benefit that some of them, particularly insoluble ones, gain better stability [100]. The NPs can be surface-coated with targeted ligands such as antibodies or antibody fragments, small molecules, peptides, or aptamers. The conjugation of the NPs with a ligand enables active recruitment of the NP, cooperatively with the passive targeting based on the EPR effect. The ligand ensures high NP specificity by lowering the percentage of positive surgical margins during tumour resection surgeries [101].

Because of their optical characteristics, polymeric NPs have been used to enhance in vitro and in vivo bioimaging. Semiconducting polymeric NPs are among the most technically advanced optical imaging probes because they exhibit excellent photostability and brightness, a large Stokes shift, and tunable optical characteristics [102]. This new formulation enabled us to overcome the difficulties associated with the free dye, such as its hydrophobicity, photobleaching and aggregation-caused quenching (ACQ) manifestations, particularly in vivo. Remarkably, recent research has shown that using bulky hydrophobic counterions in NPs improves their bioimaging performance by reducing dye leakage and ACQ [103].

Nanoformulations were created by combining hyaluronic acid (HA) with an aminopropyl-1-pyrenebutanamide (PBA), a hydrophobic ligand that induces NP self-assembly [104], to enhance the ICG's limited tumour retention and optical stability while also employing non-toxic and biodegradable polymers. The ICG molecules were physically entrapped in the hydrophobic pockets of HA-NPs and are known as NanoICG. NanoICG NPs were reported to be non-toxic and biodegradable. Their outstanding nano-morphology increases circulation time, facilitates delivery to tumours, ensures better cellular internalization, and improves intra-operative contrast and surgical advantages in solid tumours such as pancreas, breast, liver, and prostate. Preclinical investigations of prostate cancer xenograft models yielded promising results, and intravenously administered NanoICG improved tumour signal-to-noise ratio, at 24 h, by 2.9-fold compared to free ICG [104–106].

Recent fluorescence-imaging techniques employes dyes that absorb near-infrared-II (NIR-II) light (1000–1700 nm) with remarkable penetration depth, less photodamage, and low autofluorescence within the extended spectral region [102, 107]. The use of NIR-II imaging-guided surgery has the potential to improve tumour margin estimation and precision. However, many NIR-II probes have poor water solubility and slower excretion kinetics. Some formulations use PEGylation, leading to self-assembly. The HISSNPs represent an example of polymeric NPs without PEGylation in which the IR-1061, a commercial NIR-II dye, has been conjugated with hyaluronic acid chains [107]. HINSSNP is an activatable NIR-II probe and employs a “dual lock-and-key” mechanism simultaneously activated by dual-pathological tumour microenvironment (TME) parameters [107, 108].

While up-conversion NPs have been studied for decades, the down-conversion luminescence of rare-earth-based probes in the second near-infrared (NIR-II, 1,000–1,700 nm) window for in vivo biological imaging has only recently been discovered [109]. Among them, erbium-based rare-earth NPs (ERNPs) represent NIR-II probes with down-conversion luminescence in the 1500–1700 nm range of the NIR-IIb window. In a recent study, the administration of ERNPs-TRC105 probes to mice with 4T1 murine breast tumours enabled high spatial resolution during surgery, determining tumour resection margins unambiguously. The NIR-IIb imaging detected residual tumour lesions composed of a small number of cancer cells, avoiding excessive resection of non-tumour tissue and making this NIR-IIb imaging-guided surgery highly accurate up to the few-cell level [110].

The TME’s unique properties were evaluated to develop novel tumour-targeting NP formulations. The TME becomes more acidic and hypoxic and has an overexpression of proteolytic enzymes due to the high energy demand needed to support the development and survival of cancer cells. The TME's distinctive hallmarks derive from a change in the cancer cell metabolism from oxidative phosphorylation to aerobic glycolysis, known as the Warburg effect, that lowers the pH to values below 7. The pH-activatable indocyanine green-encoded nanosensors (PINS) are pH-sensitive NPs made of [poly (ethylene glycol)-b-poly (ethyl propyl aminoethyl methacrylate)] (EPA) copolymers with several repeated EPA monomers and ICG dye units. When the PINS nanosensors reach TME with pH values below 7.0, they are activated, disassociating themselves into individual protonated unimers, amplifying tumour signals and allowing deeper fluorescence penetration in tissues [111]. At pH 7.0 the PINS remain intact NPs with no fluorescence signals. Preclinical investigations revealed that PINS enables superior contrast compared to a panel of NIR imaging probes frequently used and is effective in orthotopic and metastatic models. Zhao and colleagues demonstrated that the PINS use allows for 100% sensitivity and 100% specificity, ensuring efficient debulking surgery and complete survival in models of head and neck cancer and small occult breast nodules. Due to the promising preclinical results, the PINS nanosensors (referred to as Pegsitacianine, or ONM-100) were administered in patients with solid tumours such as breast cancer, head and neck squamous cell carcinoma, colorectal cancer, prostate cancer, ovarian cancer, urothelial carcinoma, and non-small cell lung cancer undergoing routine surgery, and the clinical trial (NCT03735680) completed phase 2 on February 1, 2022 (Table 1).

### Inorganic nanoparticles

Gold, metal oxide (Fe3O4, WO3, WO2.9), semiconductor nanocrystals (quantum dots, QDs), and silica NPs are among the inorganic NPs investigated for image-guided surgery. Inorganic NPs offer several advantageous characteristics, including ease of manufacturing, variable size, heat or reactive oxygen species (ROS) generation, x-ray absorption, and energy transfer capabilities [112].

Because of their advantageous properties, such as biocompatibility and ease of modification, gold NPs (AuNPs) are among the most commonly employed inorganic NPs in many fields [112, 113]. Their surfaces can be readily modified, with various agents such as polymers, drugs, contrast agents, antibodies, and proteins. AuNPs have tunable surface plasmon resonance due to their size, shape, and structure. As a result, they have significant fluorescence absorption caused by resonance energy transfer (FRET) and nonradiative energy dissipation capabilities that can create heat energy through electron excitation and relaxation. For increased stability, the surface of AuNPs has been modified using biocompatible materials such as polyethylene glycols (PEGs) and glycol chitosan (GC).

Several therapeutic AuNPs have entered clinical studies [114]. These nanoconstructs have specific critical properties that make clinical translation easier. Unlike other multifunctional NPs described in the literature, clinically investigated AuNPs have basic compositions, and in vivo behaviour is easier to predict and manufacturing easier to scale up [114]. On the other hand, the concerns about the long-term accumulation of gold in the organism require that the advantages of AuNP-based treatments clearly exceed the potential risks. There are currently no clinical trials using AuNPs in image-guided surgery, but numerous preclinical investigations in this field. In a recent study [115], adding small biocompatible ligands to the gold core, such as zwitterionic or pegylated moieties, enhanced the optical characteristics of the AuNPs. Besides, it prolonged their plasma half-life while preserving renal clearance. Functionalized AuNPs indicated a tumour-to-healthy tissue ratio greater than 1.5 within the first hour of administration, allowing for rapid and extended tumour visualization. In another study, a multi-functionalized PEG-AuNP-Cy5.5-anti-EGFR antibody was employed, to accurately guide endoscopic near-infrared photothermal treatment [116]. This study proved the efficiency of multifunctional AuNPs in the theranostics of upper GI cancer and proposed a novel and viable design for real-time in vivo application via endoscopy. The anti-EGFR antibody was the ligand of choice on AuNPs since EGFR is overexpressed in multiple cancers. Although competition binding with other EFGR-expressing tissues is predicted, directing functionalized AuNPs to tumour areas utilizing a synergistic mix of active targeting (antibody-driven) and passive accumulation (EPR effect) increased the number of AuNPs within the tumour lesion. A recent preclinical investigation showed that the AuNPs functionalization with the cmHsp70.1 antibody, is a promising approach for tumour targeting in vivo [117]. The concentration of the probe within tumours was adequate for spectral-CT imaging, allowing for 3D reconstructions and quantifications.

Superparamagnetic iron oxide NPs (SPIONs) have particular relevance because they have been approved for human use by the FDA and utilized in clinics. SPIONs are a promising probe for MRI of brain tumours because they function as a negative-contrast agent, increasing image contrast by decreasing the T2 MRI signal [118]. Relaxivity may be improved further by adjusting the core size and coating. SPIONs ease of modification allows surface modification with antibodies, peptides, and polymers to give long-circulation and target-specific imaging. Currently, clinical investigations with SPIONs primarily include the development of hyperthermia. Although recent developments in preclinical research show promise for the future, clinical applications are still developing gradually [118]. Among the studies reporting promising applications in image-guided surgery, Winter and colleagues showed that after intraprostatic SPION injection, magnetic resonance scintigraphy offers a roadmap for intraoperative magnetometer-guided SLN identification and can be used to describe a valid lymphadenectomy template [119]. In a recent study, ICG was added to the SPIONs, allowing the method to be refined using integrated fluorescence imaging in a porcine model [120]. A unique imaging and navigation modality for the intraoperative detection of SPION tracers was successfully created and assessed. It might broaden the options for guiding during magnetic SLN operations for several oncological reasons (e.g., penile, breast, melanoma, head-and-neck, prostate, and vulva cancer).

QDs are semiconductor nanocrystals with diameters ranging from 2 to 10 nm, high quantum yields, photostability, and tunable emission wavelengths. Because of their narrow emission peak and absorption spectra ranging from UV to visible wavelengths, multiple QDs may be triggered simultaneously under UV stimulation, enabling multicolour fluorescence imaging. Furthermore, QDs can outperform single-molecule dyes in photoactivation and ROS generation because of their high photostability. Traditional QDs, on the other hand, are composed of heavy metal components such as Cd2 + and Pb2 +. The cytotoxicity of the heavy metal ions produced and their potential risk in biological systems restrict the theranostic applications of QDs.

A novel generation of near-infrared fluorescent core-shell silica-based NPs (Cornell prime dots) tuned to hydrodynamic diameters of 3.3 and 6.0 nm was successfully created, with improved photophysical properties over the parent dye [121]. A neutral organic coating reduced serum protein adsorption and promoted effective urine elimination. Molecularly targeted Cornell prime dots entered clinical trials, as reported in Table 1.

Among the most significant examples of inorganic NPs at the preclinical stage is a Gd-based degradable hollow virus-like NP made using a hard template of mesoporous SiO2 with loading ICG [122]. The NP was subsequently surface-modified with cyclic RGD pentapeptide, thus combining multimodal imaging with tumour‐targeting strategies. The nanoprobe significantly increased tumour retention, target selectivity, and water stability of ICG. It also produced remarkable magnetic resonance and second near-infrared (NIR-II) window multimodal imaging in vivo. In another study, biocompatible tantalum oxide/silica core/shell NPs with multifunctional imaging and adhesion properties have been developed. These characteristics allowed this formulation to be effectively used as an immobilized marker for image-guided procedures and as a hemostatic adhesive for minimally invasive procedures.

### Lipid nanoparticles

Studies involving lipid NPs in image-guided surgery gave encouraging results, albeit still in the preclinical stage. Liposomal formulations of ICG provide the ideal properties for imaging lymphatic function: (i) they improved optical properties and long-term fluorescence stability in solution; (ii) following intradermal injection, numerous draining lymph nodes can be seen clearly, allowing the dynamics of lymphatic flow to be measured [123]. A hereditary model of lymphatic malfunction and a B16 melanoma tumour model of lymphatic metastasis was used to confirm further quantitative imaging of lymphatic function using the liposomal formulation of ICG. A subsequent study attempted to re-engineer the PEGylated liposomes intended for systemic blood circulation and tumour imaging [124]. By utilizing the photoacoustic effect to overcome the limitations of pure optical approaches, photoacoustic tomography, which has a high spatial resolution and deep penetration (up to 7 cm), provides a new potential in image-guided surgery. Based on the effectiveness of clinically-applied PEGylated liposomes, encapsulating indocyanine green as an optoacoustic agent was demonstrated [124]. ICG-loaded gold nanorod-liposome core-shell NPs were developed to integrate photoacoustically and fluorescence imaging strategies [125] in another multimodal approach. The tumour detection effectiveness and surgery guidance were demonstrated in orthotopic liver cancer mouse models.

Non-lamellar lyotropic liquid crystalline nanocarriers called exosomes and cubosomes are lipid-based NPs that can be specifically engineered to meet precise therapeutic needs in nanomedicine [126]. A growing number of studies have drawn attention to intriguing properties that make them good candidates for usage as nanocarriers for therapeutically active chemicals or imaging probes [37, 127–129]. However, their potential as nanomedicines are questioned due to essential aspects such as pharmacokinetics, hemocompatibility, toxicity, and transport properties that have not been thoroughly characterized.

### Nanosized ultrasound contrast agents

Ultrasound contrast agents are generally classified into three types based on the composition of their cores: gas, solid, and liquid [130]. Ultrasound contrast agents with a gas core cause substantial acoustic impedance variation within tissue interfaces and can create the highest acoustic intensity among the other classes. Several microbubble ultrasound contrast agents, including Definity, Echovist, and Sonovue, have received clinical approval. It is worth mentioning that there is a significant link between particle size and acoustic echo reflectivity. On the other hand, nano-bubble gas-core ultrasound contrast agents can escape from the RES, enter the tumour tissue through the endothelium gap, and accumulate at the target site. As a complement to microbubbles, nanosized ultrasound contrast agents are developed as contrast enhancers for ultrasound molecular imaging with the capacity to permeate vasculature for extravascular imaging [131]. A significant accumulation of nanosized ultrasound contrast agents in an investigated area can improve the signal of target regions while increasing persistence time, particularly in malignancies with the EPR effect [132] when compared to microbubbles. Nano-bubbles have a gas core with a coating layer that includes lipid, protein, and polymers [e.g., poly-lactic-co-glycolic acid (PLGA), poly-lactic acid (PLA), and polyethylene glycol (PEG)] to optimize the structure’s stability. Furthermore, numerous tactics have been implemented to maximize the nano-bubbles stabilities, including heavy gas core and conjugation, with a targeting agent [133, 134].

Because of their low impedance, liquid-core ultrasound contrast agents provide poor contrast enhancement due to low acoustic dispersion inside the arteries. In this regard, liquid-based compounds such as perfluorocarbon (PFC), including perfluoro-pentane (PFP), perfluorooctyl bromide (PFOB), and perfluorohexane (PFH), have created a remarkable echo in preclinical studies when they concentrate in the target tissue/cells or change phase from liquid to gas by applying thermal energy [135]. Nanodroplets are ultrasound contrast agents that have a liquid core with a low boiling point and are enclosed in an organic shell. They can change from a liquid to a gas phase when heated. They're also referred to as phase-change droplets [136]. Liquid fluorocarbons can become micron-sized bubbles under ultrasonic irradiation or other energy delivery, which improves their effectiveness in ultrasound imaging and treatment.

A noninvasive thermal procedure called high-intensity focused ultrasound (HIFU) has recently shown much promise for treating tumours. Applying targeted ultrasonic waves to the tumour tissue so that the tissue heats up and becomes necrotic is the physical principle behind this interventional method. In 1942, Lynn et al. introduced the idea of HIFU-assisted noninvasive surgery [137]. Nano-biotechnology has now been introduced into HIFU cancer surgery research. PLGA allowed PFP encapsulation to produce a nanoemulsion in a recent preclinical investigation. Phase-shift PFP nanoemulsions could enhance pulsed HIFU ablation in the subcutaneous xenograft rabbit model [138].

Because of their distinct properties, solid-core nanosized ultrasound contrast agents have been developed for ultrasound contrast enhancement. These properties include increased stability and suitable acoustic impedance variations between solid-core materials and soft tissue. The solid-core nanosized ultrasound contrast agents provide a more robust reflectivity output, a better signal-to-noise ratio, and increased contrast enhancement. These NPs have strong, intravascular and within-soft tissue echo intensities [139]. As a clinical application example, Fand and colleagues utilized surface-modified Fe3O4 magnetic NPs to create magnetic lipid ultrasonic microbubbles (MLU-MBs) using the mechanical oscillation method with polyethylene terephthalate (PET) [140]. MLU-MBs could improve the accuracy of ultrasound localization-guided breast-conserving surgery for tumour removal in a cohort of 92 patients undergoing breast-conserving surgery for breast cancer.

The following are the primary issues in studying nanosized ultrasonic contrast agents. The decline in signal strength, the decrease in the inner diameter of contrast agents, and the loss in backscattering ability are all significant issues to be addressed in contrast agents’ nanosized ultrasound research. It is challenging to precisely manage the size of nanosized ultrasound contrast agents that cross the vasculature and scatter strongly. Furthermore, their shell and core will affect the nanosized ultrasound contrast agents’ stability and residence time. Thus, future studies will focus on how to lower the size of nanosized ultrasound contrast agents while increasing the signal [131].

### Challenges and future perspectives

There is a broad range of sensitivity, spatial resolution, and accessibility in the imaging technologies discussed. Image-guided surgery enables the differentiation of pathology from nearby normal tissue, which improves resection accuracy [3, 10, 23]. Moreover, the intraoperative imaging procedures can be developed to enable lymphatic mapping of tumour-draining lymph nodes and target the analysis of regional nodes at risk for metastases [27, 59, 91]. However, the true potential of image-guided surgery may lie in contexts where radical excision of microscopic or diffuse infiltrative lesions is necessary with acceptable safety margins. The surgical process of removing an incurable tumour is known as “debulking” or “cytoreduction.” It is further classified as complete or optimal, with the “complete” indicating the absence of any visible residual tumour and the “optimal” describing a maximum residual tumour size of 10 mm. These indications present the greatest challenges for the use of image guidance because they call for the development of tracers with high affinity and specificity for cancer tissue as well as the generation of target-to-background contrast that allows accurate detection with the chosen instrumentation [11].

Typically, radiotracers are superior to optical tracers because they provide stronger signals with less interference. For applications requiring minimal signal attenuation and scattering (i.e., a high penetration depth), X-ray and MRI are the optimal modalities, followed by radiotracer-based detection, ultrasound, and then optical tracers. Thus, the applications of optical imaging are still limited to superficial examinations, where the effects of light attenuation from both the emitted signal and the background light can be minimised. In addition to high sensitivity, detecting small lesions requires an imaging modality with high spatial resolution. When compared to other methods, optical technologies outperform in this area [14]. Moreover, the availability of chemical dyes with a range of spectral properties makes multicolor imaging approaches possible with fluorescence imaging [36]. Given the complementing properties of the various imaging modalities, we believe that hybrid techniques based on the integration of multiple methodologies will provide the best outcomes in the future [10, 16].

Improved knowledge of molecular pathology has revealed an extensive range of options for image-guided surgery. The identification of unique cancer cell markers not only drives the creation of novel precision medicine trials but also paves the way for novel molecular imaging strategies. Therapeutic antibody labeling, for instance, has previously been established in ongoing surgical studies as a means of translating therapeutic biologicals into imaging agents [13]. Certainly, in this context, the future challenges will lie in identifying relevant imaging biomarkers.

NPs have an important impact on cancer imaging due to their ability to facilitate the generation of multimodal contrast agents and surface functionalization for precise molecular recognition [15, 16, 101, 141]. However, two crucial factors should be addressed when analysing the gap between the numerous preclinical studies and the small number of clinical studies. Is the configuration of the NPs chosen to maximise their accumulation in the tumour? Second, how can we conduct an accurate screening to determine which patients are candidates for nanomedicine? Both factors are critical in determining whether a nanotherapeutic will be effective.

Due to the EPR effect, the leaky vasculature of tumors promotes the passive diffusion of NPs [82, 142]. Beyond the EPR effect, a number of strategies are under investigation for accumulating NPs in the desired lesions [15, 83]. At present, important goals have been achieved in preclinical studies on actively targeted NPs, with intriguing results arriving from the clinical trial of the integrin-targeting Cornell prime dots applied in real-time intraoperative optical imaging guidance [91]. In addition, targeting the tumor microenvironment (TME) is a promising new approach in clinical testing. ONM-100 is an NP-based fluorescence imaging agent that reacts to pH changes caused by cancer acidosis; recent research showed that it was well tolerated and that four solid tumor types could be detected in- and ex vivo in thirty patients [92]. Undoubtedly, additional preclinical research and clinical trials will be required to improve nano-based imaging contrast agent approval and clinical translation [141]. NPs have unique properties that offer enhanced imaging sensitivity and specificity, which could have a significant impact on healthcare and patient outcomes.

## Conclusions

Over time, technologies will almost certainly transform the surgical approach to cancer. Indeed, technological advancement in the field of molecular imaging has been remarkable and promising. However, many steps must be taken to progress from proof-of-concept studies to routine clinical use. In particular, we must ensure that clinical requirements keep pace with tracer chemistry, device physics, and the increasing digitization of the operating room. Reaching consensus through well-defined efficacy studies will help to standardize all aspects of the imaging cascade. Many trials are currently underway, and the results will reveal the actual benefits for patients, especially in terms of survival. Unfortunately, the time frame will not be brief. Besides, the costs of technology must be proportional to the benefits. Technology diffusion may be challenging in non-hyper-specialized centres, but centralization might overcome this issue. Which scenario will emerge in the future: will technology drive clinic evolution, or clinics drive technological advancement?
